# The role of a national evaluation system in promoting dairy sustainability*

**DOI:** 10.3168/jdsc.2024-0645

**Published:** 2025-01-10

**Authors:** Asha M. Miles, Kristen L. Parker Gaddis, John B. Cole, Robert H. Fourdraine

**Affiliations:** 1 Animal Genomics & Improvement Laboratory, Agricultural Research Service, USDA, Beltsville, MD 20705; 2 Council on Dairy Cattle Breeding, Bowie, MD 20716; 3 Department of Animal Sciences, Donald Henry Barron Reproductive and Perinatal Biology Research Program, and the Genetics Institute, University of Florida, Gainesville, FL 32611; 4 Department of Animal Science, North Carolina State University, Raleigh, NC 27695; 5 Dairy Records Management Systems, North Carolina State University, Raleigh, NC 27603

## Abstract

Sustainable agriculture is best defined as a balance of practices that promote economic vitality, protect the natural environment, and build healthy communities in the present without compromising the future. The dairy industry has achieved tremendous gains in productive efficiency following decades of work with these goals in mind. A major tool contributing to this progress is the US National Cooperator Database (NCD), developed in partnership with key industry groups. Involving a complex system of both domestic and international data sharing, the NCD now includes >9 million genotypes, >100 million pedigrees, and >100 million lactation records. The primary outputs of this system are national genetic and genomic evaluations, published tri-annually. Greater genetic gains have resulted in faster rates of inbreeding, a well-established antagonist to cow health and performance. Dairy genetics are traded all over the world, and the NCD enables the monitoring of inbreeding at the global population level. Dairy systems are continually evolving thanks to technology advancements, changing consumer values, and resource availability; frequent interrogation of the NCD can facilitate the early detection of changing industry trends that affect the accuracy of existing evaluations, but also highlight the need for improvement tools that are just as dynamic as the dairy industry. An industry-wide effort is underway to develop a herd-level sustainability metrics platform that would support dairy producer ability to farm and track their progress in key health and production areas. This pre-competitive collaboration among dairy industry groups ensures the reliable flow of accurate data so we can continue to develop tools to support the dairy producer in the present and future.

## Highlights

• Over a century ago, the United States began centralizing dairy herd information records.
• This database is key to monitoring trends and promoting genetic progress.
• Novel management and genetic tool development are possible.
• Modernizing data collection systems will be critical to their continued impact.

## Graphical Abstract

**Figure:**
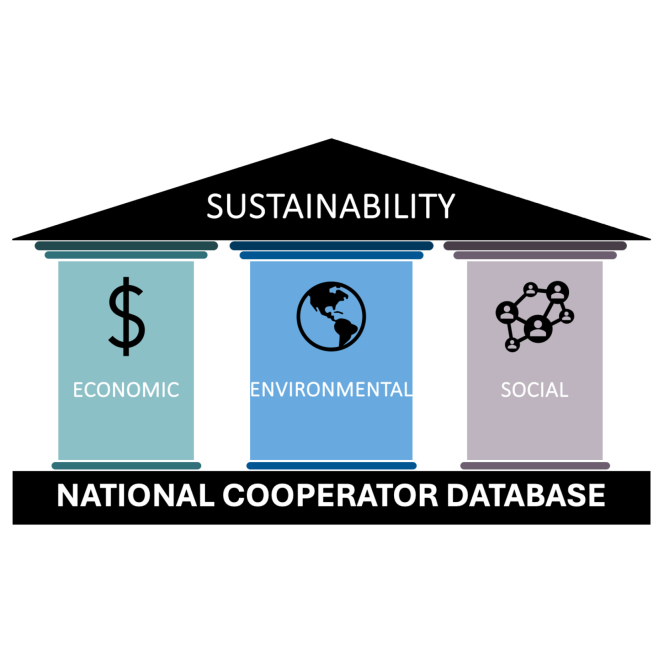
**Summary:** In this article, we describe the establishment of the National Cooperator Database, the largest animal database in the world, and how this tool is an essential foundation for promoting sustainability from a dairy breeding perspective. We discuss 3 questions: (1) How have we been addressing sustainability in US dairy breeding? (2) What are the opportunities? and (3) What are the lessons from the last century of US dairy breeding programs? Major areas of opportunity include preserving genetic diversity by managing the increased rate of inbreeding observed in the US dairy population and modernizing data collection systems to enable the use of high-throughput phenotyping data for the evaluation of new economically important traits. In addition, we describe original research to establish a herd-level sustainability metrics tool through a consortium of partners committed to pre-competitive collaboration.

What constitutes sustainable agriculture was explicitly enumerated in the “Farm Bill,” written into law in 1990 (Public Law 101–624, Section 1603; p. 104, Stat. 3706), and described as follows:

Sustainable agriculture [is] an integrated system of plant and animal production practices … that will, over the long term: satisfy human food and fiber needs; enhance environmental quality and the natural resource base upon which the agricultural economy depends; make the most efficient use of nonrenewable resources and on-farm resources and integrate, where appropriate, natural biological cycles and controls; sustain the economic viability of farm operations; and enhance the quality of life for farmers and society as a whole (Food, Agriculture, Conservation, and Trade Act, 1990).

This emphasizes classical definitions of sustainability as a concept upheld by 3 pillars concerning (1) promoting economic vitality, (2) protecting the environment, and (3) building healthy communities. Remove any pillar and the system is out of balance and no longer sustainable. With this framework in mind, in this article we discuss 3 questions: (1) How have we been addressing sustainability in US dairy breeding? (2) What are the opportunities? and (3) What are the lessons from the last century of US dairy breeding programs?

Arguably, the dairy industry has been very good at sustainability for a very long time, but under the brand of productive efficiency. In 1950, the United States produced ∼53 billion kg of milk from ∼22 million cows and last year in 2023 produced ∼103 billion kg of milk from ∼9 million cows (National Agricultural Statistics Service, 2024). Over 70 yr, US dairy producers doubled the amount of milk shipped while cutting the number of cows needed by over half. This is explained by the steady decrease in the number of US cows and reciprocal increase of milk produced per cow (Figure 1). In a review of changing environmental impacts of the US dairy industry between 2007 and 2017, Capper and Cady (2020, p. 10) reported that, “although U.S. [energy-corrected milk] production increased by 24.9% in the decade from 2007 to 2017, the total [greenhouse gas] emissions arising from this production increased by only 1.0%.” The question to emerge is, how are we now feeding more people with fewer cows and less environmental impact? Among many contributing factors, including improved nutrition and management, the creation of a national genetic evaluation system has been a major asset.

**Figure 1:**
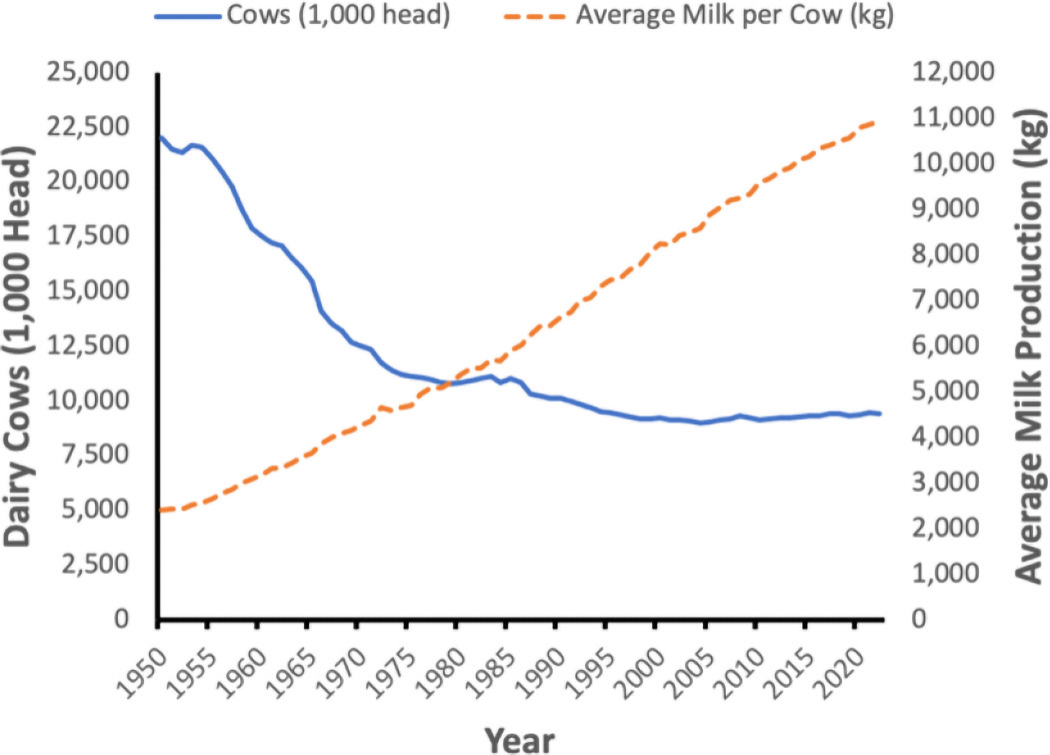
Number of dairy cows in the US national herd (solid blue line) and average milk production (broken orange line) trends from 1950 to 2022 (National Agricultural Statistics Service, USDA).

This effort began in 1908, when USDA's Bureau of Animal Industry organized cow testing associations nationally to facilitate accurate, credible, and uniform milk recording that can be integrated into a single system. By 1927, cow testing associations were renamed Dairy Herd Information Associations (DHIA) to reflect the vast amount of herd management information being collected. As early as 1915, some bull associations calculated daughter-dam differences internally, and this was popular enough that in 1926 USDA calculated and distributed sire evaluations for the first time, for just 23 bulls (Graves, 1926). At that time, 305-d lactation records were hand or typewritten and mailed to USDA on a prepaid postcard. By 1946 the USDA's Bureau of Animal Industry employed nearly 100 people to process and maintain the library of incoming paper lactation records and outgoing evaluations. Sire evaluation books were printed and distributed for nearly 40 yr, but after 1975 distribution was paperless via computer tapes, and since 1997 all information exchange has been purely electronic via the internet.

Data exchange was not the only thing to evolve over the years. The daughter-dam difference was the statistical method used by USDA until 1962 when sire evaluations were first computed with herdmate comparisons to account for different management practices. Two years later, national evaluations became the standard, replacing the different evaluations offered by various regional processing centers. These changes paralleled evolution in reproductive management; the increasing popularity of artificial insemination amplified the rate of germplasm exchange across the country, making this standardization key to enabling fair comparisons. Consequently, in 1989 the current “animal model” was implemented, which considers relationships among all cows and bulls, not just herdmates. Following the genomics boom of the early 2000s, official genomic evaluations were computed in 2009. A full accounting of this history is available from VanRaden and Miller (2008). The scale of this effort became such that in 2013, responsibility for the administration of evaluations was turned over to the newly reformulated Council on Dairy Cattle Breeding (**CDCB**), and the Bureau of Animal Industry, now called the Animal Genomics and Improvement Laboratory, remains as their research partner.

Today's evaluation system looks quite different than the very first DHIA, established in 1905 in Newaygo County, Michigan. Data come to CDCB from regional dairy records processing centers (**DRPC**), breed associations, the National Association of Animal Breeders, genomic nominators, and genomic laboratories (Cole et al., 2021), and even foreign data from 36 countries are transmitted by the Interbull Centre (Uppsala, Sweden). These records are heavily edited for quality assurance and stored in the National Cooperator Database (**NCD**), which currently holds >100 million lactation records, >100 million pedigrees, and >9 million genotypes, making it the largest animal database in the world. One of the tools to come out of this system is the Lifetime Net Merit (**NM$**) Index, a profit function that ranks animals based on their combined genetic merit for economically important traits and estimates how much lifetime profit they will transmit to their progeny (VanRaden et al., 2021). Tracking genetic trends for NM$ reveals a steady increase in genetic gains over time and steeper slopes corresponding to faster gains since incorporating novel technologies like genomics (Figure 2; CDCB, 2024a). A recent study validated traits in NM$ by associating genomic evaluations for heifers at birth with their eventual performance, finding that genomic evaluations add substantial accuracy and value (Toghiani et al., 2024). Most traits in NM$ are also reflected in sustainability priorities identified by other countries (ICAR, 2024), reinforcing the idea that productive efficiency runs parallel to sustainability.

**Figure 2:**
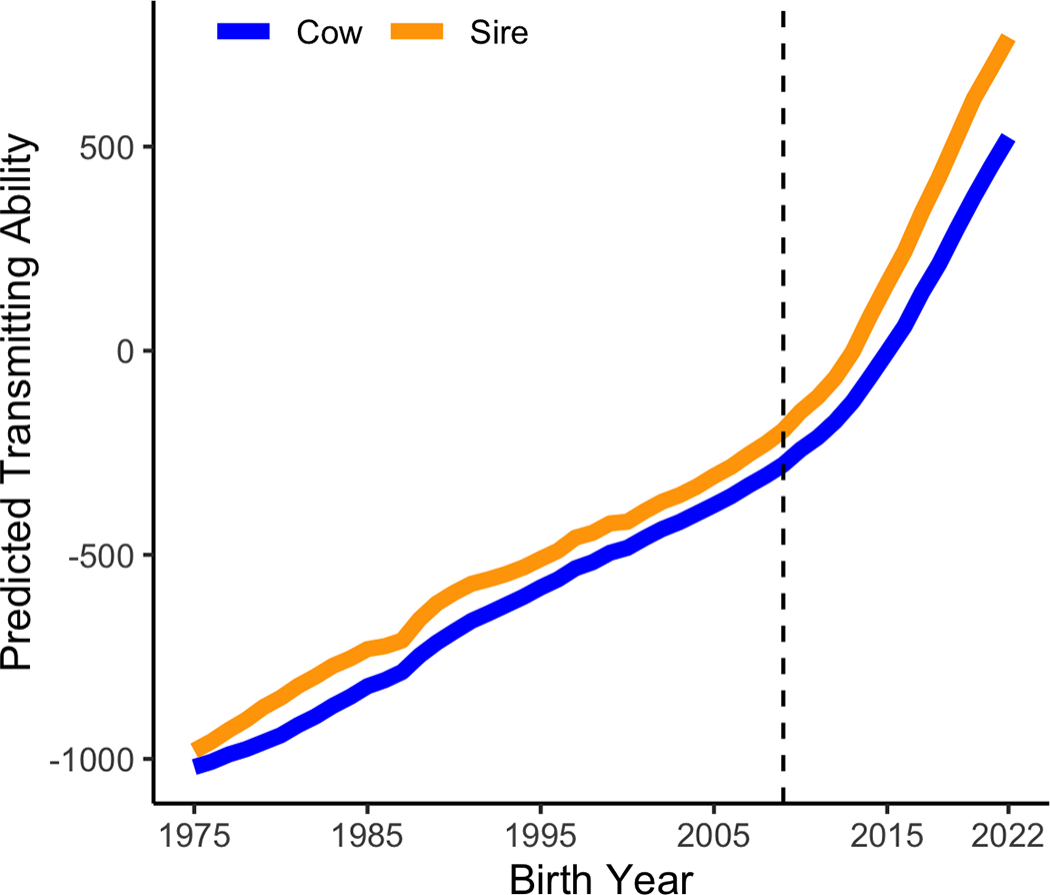
Genetic trends for Lifetime Net Merit in bulls (orange line) and cows (blue line) born between 1975 and 2022.

Both animal breeding and sustainability involve long-term planning for the future, which requires accounting for changes in climate, consumer values, and socio-political dynamics out of our control that affect agricultural systems. Acknowledging that our world is one of disruption, change, and uncertainties means we should focus on priorities that are relevant independent of changing conditions.

Higher rates of inbreeding are an inevitable consequence of selection in finite populations and have accompanied the genetic gains observed since the implementation of effective genetic improvement programs. Genomic selection has provided additional increases in both selection intensity and rates of inbreeding (Guinan et al., 2023). Indeed, annual Expected Future Inbreeding (**EFI**; the average inbreeding expected when a bull is randomly mated to the population) was <1% in 1960 for Holstein, increased steadily to ∼6% by 2009, and reached 7.5% in 2023 (CDCB, 2024b) following 15 yr of genomic selection. The concern is potential inbreeding depression: an overall decrease in fitness due to increased genetic load that results from recessive deleterious mutations and partial directional dominance (Maltecca et al., 2020). Inbreeding does not cause genetic defects, but can reveal them when a popular bull is a carrier of a condition like HH1 because of increases in carrier frequency (e.g., Cole et al., 2013). With dairy genetics traded all over the world, the NCD enables the monitoring of inbreeding and harmful haplotype frequencies at the global population level.

While inbreeding is unavoidable in a finite population, it can be managed by introducing new alleles into the population. “Population” is a loosely defined term; for closed herds this can be as simple as opening the herd to new genetics, but even the global dairy population has become highly genetically similar. Due to the heavy use of popular sires, all contemporary Holstein lineages trace back to 2 bulls from the 1960s: Pawnee Farm Arlinda Chief and Round Oak Rag Apple Elevation (Yue et al., 2015). Preserving the genetic diversity we have can be done through outcrossing (choosing mates that are not closely related or within the same family lines) or crossbreeding with other dairy breeds. However, many cows are now mated at random to a portfolio of bulls due to the precision breeding challenges that come with large herd sizes. A major conundrum in the management of inbreeding is that high genetic merit bulls have high marketability, and that lower inbreeding results in slower genetic gains. Sustainable dairy breeding means balancing genetic diversity goals with preserving economic viability of both farms and studs.

For an example of how inbreeding can become a big problem, we look to other food production systems that are highly specialized and genomically homogeneous. A small genetic base may translate to a fragile population that cannot respond well to environmental or management changes. A timeline of disease outbreaks in highly specialized systems shows occurrence at a much more rapid rate as systems become less diversified (IPES-Food, 2016). If we continue in this way, the dairy population will start to look like swine and poultry breeding—an extremely homogeneous population—but without the controlled environments needed to protect them.

The International Committee on Animal Recording (**ICAR**) provided a harmonized approach to assessing dairy herd sustainability using quantitative measures routinely collected through milk recording programs. The results of their efforts are a standardized list of 43 traits representing the 5 categories of Feeding and Production, Fertility, Health, Longevity, and Young Stock (ICAR, 2024). Instead of outlining a sustainability index, ICAR encourages countries to develop their own tools using these guidelines. A consortium of NCD partners are actively working on implementation of a herd-level sustainability metrics platform in the United States.

In a proof-of-concept project, DRPC provided herd-level data for average DIM (n = 10,003 herds), average calving interval (n = 9,905), average SCC (n = 9,830), average culling age (n = 10,041), and average age of first calving (n = 10,095). Peer groups were defined according to 3 criteria: (1) dairy breed, (2) herd size (small <250, medium 251–999, and large >1,000 milking cows), and (3) climate regions ranging from 1 (hottest) to 5 (coldest) (Miles et al., 2023b). There are 443 Holstein herds of medium size in region 4, and the percentile ranking of a randomly selected herd from this peer group is shown in Figure 3a. Producers may use this as a benchmarking tool, seeing what is achievable by herds in their peer group. Most existing sustainability metrics or indexes default to percentile rankings, but this picture of herd performance should be expanded. Figure 3b shows the percentile ranking for this herd for SCC overlaid on a histogram for all herds in their peer group (32%, thick black line). The area under the curve is shaded not by percentile ranking but by whether the actual value for the trait falls within desired margins. Percentile rankings are inherently limited—someone must be last, and a herd could have an acceptable SCC but low percentile ranking depending on peer group performance. The next challenge is reaching industry consensus on optimal ranges for each trait. This tool will be implemented as a web-based application that is released confidentially to herd owners to use internally for management or externally for potential premiums on sustainably produced milk, at owner discretion. At this point we are not recommending a sustainability “index” because traits of inclusion or potential weights are difficult to standardize across varied production systems. This is the same reason ICAR did not give recommendations for an index, preferring to leave that to the judgment of the users. Efforts are ongoing to expand this to as many of the 43 ICAR sustainability traits as possible (some are not routinely collected in the United States) and to enable continuous data flow for longitudinal performance tracking. Figure 3c demonstrates that this particular herd is performing in a steady state concerning SCC, based on data available from October 2023 to April 2024. There is not a straight line from these traits to the big picture of “sustainability,” which is much more complex than improved cow performance and requires consideration of factors like animal welfare, food safety, supply chain management, and soil and water quality impacts largely resulting from farm management practices. Dairy producers need social license to operate, and improving farm management and expanding selection tools will not necessarily translate to consumers accepting that we are being responsible in our stewardship. Community engagement remains critical, and even more important as the average person becomes farther removed from agriculture.

**Figure 3:**
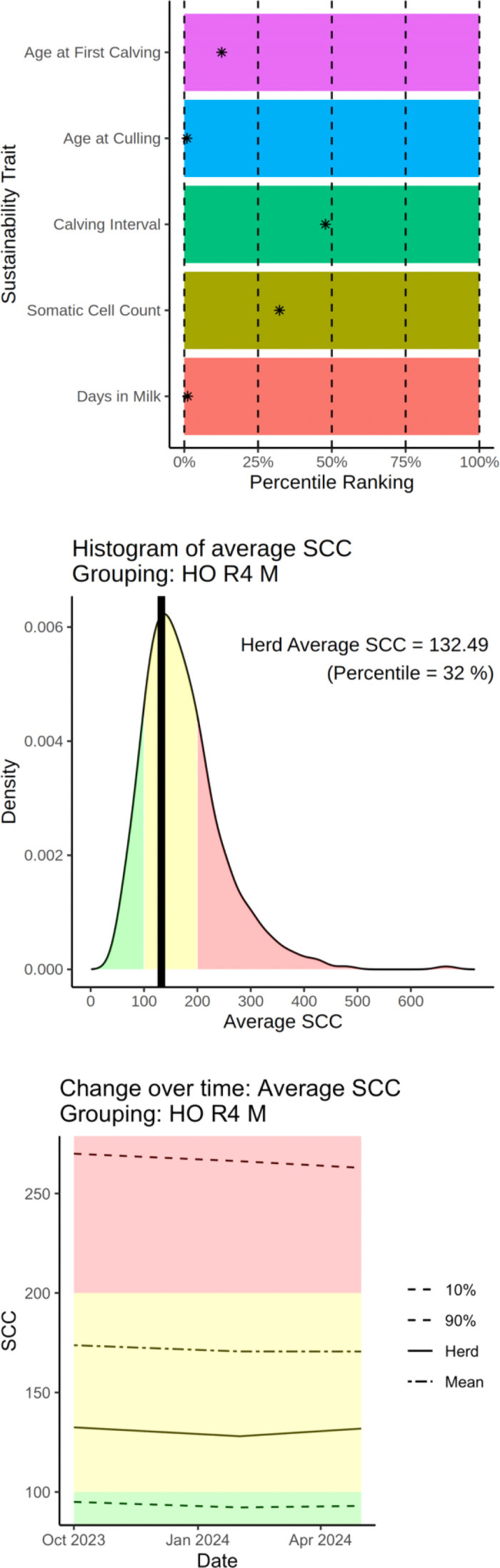
Proposed herd-level sustainability metrics platform where herds are given a percentile ranking for each trait (*), and they can expand this information to see the density distribution for herds in their peer group, and track their progress over time. HO = Holstein; R4 = region 4; M = medium herd size.

There is recent interest in new traits encompassing issues like climate resilience, methane emissions, animal welfare, and performance in nonconventional systems. Many researchers want to work on traits that are likely to be economically important, but producers are not incentivized to contribute data because the data will not directly affect farm profits (Cole et al., 2020). Incentives must be shifted for contribution of data related to these sensor-based systems to emerge beyond the margins. Several initiatives are underway to expand the collection of data related to animal welfare (e.g., hoof health) and climate impacts (e.g., methane emission). This involves partnership with university research herds to assemble a reference population of high-quality and difficult-to-measure phenotypes that can be extrapolated to the larger dairy population through genomics. Selection can only improve what we can measure, and as dairy production systems become increasingly complex, so do the requirements of accurately measuring animal performance. Enter precision agriculture. Precision livestock monitoring technologies primarily take the form of economically justifiable sensor systems that can be wearable (i.e., boluses; neck, leg, or ear tags) or mounted (i.e., a stationary camera, robotic milking system; Halachmi et al., 2019). Autonomous, sensor-based phenotyping represents a huge opportunity to increase the number of traits that can be improved through genomic selection; a caveat to their use is that they were designed to prompt management outcomes and are not necessarily available or appropriate for research use.

The traditional (non-sensor-based) data of the last 100 yr have not been without error or bias, but progress was still achieved through rigorous editing, the establishment of data quality standards, and the ready cooperation of records processors and providers. With the exception of some production traits, there are no standard practices for users or validation, maintenance, and calibration protocols for these novel sensor data, creating substantial system bias and individual sensor bias. Because these systems are designed to function as a management tool, data are only stored for a short time before they are overwritten. Data storage, flow, quality control, and quality assurance standards need to be established before they can be used on a national scale, and even their on-farm use should be interpreted with caution. At this time, no standards exist for sharing sensor-generated data, which may limit their use and benefits. Due to the proprietary nature of sensor technologies, many companies have implemented restrictions on the granularity and amount of data they are willing to share and may require a fee for exchange of this data. That philosophy contradicts our current state of affairs where CDCB serves as a steward of farm data, but sole ownership and rights pertaining thereto remain with the producer. This issue of sensor data ownership is evocative of recent controversies surrounding technology companies both within and outside of the agricultural sphere, backed by the “right to repair” movement. Extended Use License Agreements are complex and rife with legalese that limits the use of equipment; these are signed at time of purchase and users often do not fully understand their ramifications. Frequent software and technology updates create problems for so-called legacy equipment as repairs and maintenance are not available for older versions. Two big questions to emerge in this data-driven age are (1) how we will standardize it and (2) who can use it. Furthermore, the transformation to purely electronic and often cloud-based systems brings new security challenges to the national system. Cyber-attacks on various providers of agricultural information services earlier this year have disrupted the functionality of data systems, but also introduced new concerns about confidentiality breaches. Suffice to say, our biggest challenges in the coming years will likely be legal, not scientific.

The future is as inscrutable to us as it was to those who founded our national system 116 yr ago. What started in an 800 square mile (1,200 square km) county in Michigan now reaches nearly every corner of the globe, with more than half of the female genotypes in the NCD originating from outside the United States (VanRaden et al., 2024). Where the very first 305-d lactation records were painstakingly kept by hand and mailed to processing centers, today electronic data are transmitted in real time. One hundred years ago, cows were mainly selected for yields; CDCB now publishes evaluations for 50+ traits related to productive efficiency, health, and welfare. In pursuit of learning from the past to plan for the future, the first question is, what has made us successful (sustainable)? The qualities echoed in every example in this article are those of flexibility, adaptability, cooperation, realism, and eagerness to evolve to meet change.

Collecting high-quality phenotypic data will continue to be key to advancing genetic evaluations and selection strategies. Sensor data represent a huge opportunity but will require further modernization of our data collection systems. As farms continue to grow and invest in more technology, staffing needs are decreasing and decision-making is highly affected. Many alerts are now generated by equipment (vs. people) and research will be required to determine their precision and accuracy, and how they can be used for selection. A good example of this is milking speed (**MSPD**). The CDCB has appointed a task force to evaluate the feasibility of a MSPD evaluation using quantitative measures generated by inline milk meters that are becoming very prevalent on large dairy herds. In a study of >40 million observations of MSPD measurements derived from individual milkings, researchers found that, among other factors, the meter manufacturer significantly influenced these measurements (Miles et al., 2023a). Manufacturers may have different definitions of when milk flow begins and ends, and this may even vary within manufacturer depending on parlor configuration, which is highly customizable by the producer. Nonetheless, with appropriate consideration of confounding bias, this group has determined that reliable genetic evaluations for MSPD are possible (unpublished data).

Successful launch of a MSPD trait will require the aforementioned flexibility, adaptability, cooperation, realism, and eagerness to evolve. Key to any healthy evaluation is routine data flow. Most popular herd and milking management software are not designed to store the required data to derive MSPD, and substantial changes will be required to harvest, clean and edit, and absorb said data into the NCD. If indeed a rising tide lifts all boats, all data providers and processors can benefit from early investment in infrastructure to collect this type of data. Furthermore, additional milking efficiency traits (e.g., cow behavior) can be derived from the same data. Milking speed is likely the first of many economically important traits to be published that relies on high-throughput phenotyping. As data types and quantities continue to expand, flexibility and evolution of computational methods will also be required for the delivery of large-scale genomic evaluations.

There is universal appeal to F. Scott Fitzgerald's conclusion to *The Great Gatsby*, “So we beat on, boats against the current, borne back ceaselessly into the past” (Fitzgerald, 1925, p. 193). But we conclude that our history is not something to be escaped; it is a strong foundation on which to stand, arming us with both experience and vision, and propelling us into the future.
